# Identification of New Resistance Loci to African Stem Rust Race TTKSK in Tetraploid Wheats Based on Linkage and Genome-Wide Association Mapping

**DOI:** 10.3389/fpls.2015.01033

**Published:** 2015-12-09

**Authors:** Giovanni Laidò, Giosuè Panio, Daniela Marone, Maria A. Russo, Donatella B. M. Ficco, Valentina Giovanniello, Luigi Cattivelli, Brian Steffenson, Pasquale de Vita, Anna M. Mastrangelo

**Affiliations:** ^1^Cereal Research Centre, Council for Agricultural Research and EconomicsFoggia, Italy; ^2^Genomics Research Centre, Council for Agricultural Research and EconomicsFiorenzuola d'Arda, Italy; ^3^Department of Plant Pathology, University of Minnesota Twin CitiesMinneapolis, MN, USA

**Keywords:** tetraploid wheat, stem rust, resistance genes, association mapping, linkage mapping

## Abstract

Stem rust, caused by *Puccinia graminis* Pers. f. sp. *tritici* Eriks. and E. Henn. (*Pgt*), is one of the most destructive diseases of wheat. Races of the pathogen in the “Ug99 lineage” are of international concern due to their virulence for widely used stem rust resistance genes and their spread throughout Africa. Disease resistant cultivars provide one of the best means for controlling stem rust. To identify quantitative trait loci (QTL) conferring resistance to African stem rust race TTKSK at the seedling stage, we evaluated an association mapping (AM) panel consisting of 230 tetraploid wheat accessions under greenhouse conditions. A high level of phenotypic variation was observed in response to race TTKSK in the AM panel, allowing for genome-wide association mapping of resistance QTL in wild, landrace, and cultivated tetraploid wheats. Thirty-five resistance QTL were identified on all chromosomes, and seventeen are of particular interest as identified by multiple associations. Many of the identified resistance loci were coincident with previously identified rust resistance genes; however, nine on chromosomes 1AL, 2AL, 4AL, 5BL, and 7BS may be novel. To validate AM results, a biparental population of 146 recombinant inbred lines was also considered, which derived from a cross between the resistant cultivar “Cirillo” and susceptible “Neodur.” The stem rust resistance of Cirillo was conferred by a single gene on the distal region of chromosome arm 6AL in an interval map coincident with the resistance gene *Sr13*, and confirmed one of the resistance loci identified by AM. A search for candidate resistance genes was carried out in the regions where QTL were identified, and many of them corresponded to NBS-LRR genes and protein kinases with LRR domains. The results obtained in the present study are of great interest as a high level of genetic variability for resistance to race TTKSK was described in a germplasm panel comprising most of the tetraploid wheat sub-species.

## Introduction

Stem rust, caused by *Puccinia graminis* Pers. f. sp. *tritici* Eriks. and E. Henn. (*Pgt*), is one of the most important diseases of wheat in many regions of the world (Leonard, 2001; Hodson, 2011). During severe epidemics, the disease can cause yield losses exceeding 50–70% in both hexaploid (*Triticum aestivum* L.) and tetraploid (*Triticum turgidum* ssp.) wheats (http://www.ars.usda.gov/). Additionally, wheat infected by stem rust can also suffer reduced end use quality and food security (Singh et al., 2005). Combinations of different stem rust resistance (*Sr*) genes were successfully introgressed into wheat cultivars worldwide since the 1950s, and this gene deployment scheme has effectively controlled the disease for many years (Singh et al., 2011). However, the discovery of a new aggressive race (TTKSK, isolate Ug99) of *Pgt* in Uganda in 1998 (Pretorius et al., 2000) threatens wheat production due to its wide virulence on over 80% of wheat cultivars worldwide (Singh et al., 2006; Yu et al., 2012; Sharma et al., 2013). Moreover, at least eight variants with different virulence patterns have been described from the “Ug99 lineage” of African stem rust races, further complicating the resistance breeding process (rusttracker.cimmyt.org).

Both qualitative and quantitative resistances have been reported in wheat against stem rust. Qualitative resistance is controlled by major race-specific genes (“*R*” genes) that are effective against some pathogen isolates, but not others. Oftentimes, this resistance is expressed throughout all growth and development stages of the plant. Quantitative resistance is usually based on multiple genes, each with a minor effect on slowing disease development by delaying pathogen infection, growth and/or reproduction. Since this type of resistance is phenotypically evident in adult plants only, it has been described as “adult plant resistance” (APR). APR may be more durable than resistance based on single *R* genes and is best identified by screening germplasm in the field. Accessions that exhibit seedling susceptibility to a suite of pathogen races, but develop rust more slowly compared to susceptible controls may carry useful levels of APR (Yu et al., 2014). To date, 58 stem rust resistance genes have been designated in wheat (McIntosh et al., 2011). In addition, several alleles conferring unique race specificities have been identified for many of these genes, resulting in a total of 65 numerically designated resistance genes and alleles (Yu et al., 2014). Of this number, at least 27 are effective or partially effective against races in the Ug99 lineage (Yu et al., 2014). However, as reported by Haile and Röder (2013), most of these genes were derived from wild relatives of wheat and are located on chromosome translocations that include large donor segments, harboring genes possibly deleterious to agronomic and quality traits, as was observed for genes *Sr22* and *Sr26* (Dundas et al., 2007; Olson et al., 2010). To enhance the utility of these genes in wheat breeding, there are ongoing research efforts to eliminate deleterious linkage drag and produce lines with smaller chromosome segments containing the resistance genes (Dundas et al., 2007; Haile and Röder, 2013). With regards to quantitative resistance, five APR genes have been designated (Yu et al., 2014). *Sr2*, identified in *T. turgidum*, is a quantitative resistance gene located on chromosome arm 3BS. It has been widely used in wheat breeding programs, providing durable APR for more than 50 years (Kota et al., 2006; Yu et al., 2011). However, *Sr2* only provides partial APR and is associated with pseudo black chaff, a trait that facilitates the selection of breeding lines carrying *Sr2* but may reduce yield, especially when expressed in the glumes (Kota et al., 2006; Mago et al., 2011).

Identification and genetic characterization of new sources of resistance and their transfer to adapted genetic backgrounds is of great importance for durum and hexaploid wheat improvement. In this context, the development of molecular markers closely linked to both minor genes, which contribute to APR, and also major *R* genes, which confer race-specific resistance, offers an alternative method for selection of resistant germplasm in the absence of pathogens and facilitates effective pyramiding of genes/QTL determining resistance (Sukhwinder-Singh et al., 2012). Two marker-based approaches can be used to identify the chromosomal location of resistance loci to stem rust. Linkage mapping is a highly effective approach for the genetic study of quantitative and qualitative disease resistance and has been used frequently in durum and bread wheat to detect resistance genes/QTL to several diseases (Bansal et al., 2008; Marone et al., 2009, 2013; Russo et al., 2012; Njau et al., 2013; Singh et al., 2013a,b). Linkage mapping can only sample a small fraction of the possible alleles existing in a population from which the parents originated. Moreover, there is a restricted number of meiotic events that are captured in a bi-parental mapping population and the genetic resolution of QTL mapping often remains confined to a range of 10–30 cM (Flint-Garcia et al., 2003; Zhu et al., 2008). A second approach for mapping resistance loci is association mapping (AM), where genotype-phenotype relationships are explored in germplasm collections or natural populations. The principle that underlies this approach is based on linkage disequilibrium (LD, or non-random association of alleles at adjacent loci) that tends to be maintained between linked loci over many generations (Neumann et al., 2010). AM can be conducted directly on relevant breeding material, thus allowing direct inference from data analysis to the breeding program. Furthermore, phenotypic variation is observed for most traits of interest and polymorphism is higher than in bi-parental populations (Yu et al., 2006; Zhu et al., 2008). In both tetraploid and hexaploid wheat, linkage and association mapping have already proven to be effective strategies for identifying marker-trait associations for agronomically important traits (Crossa et al., 2007; Maccaferri et al., 2011) including resistance to stem rust (Crossa et al., 2007; Bhavani et al., 2011; Yu et al., 2011, 2012; Letta et al., 2013, 2014; Njau et al., 2013; Singh et al., 2013a,b; Rouse et al., 2014; Zhang et al., 2014).

In the present study, a tetraploid wheat collection was evaluated for resistance to the virulent African stem rust race TTKSK under the controlled conditions of a greenhouse. A wide phenotypic diversity was observed for this trait in the tetraploid wheat association panel, and 35 chromosome regions associated with resistance were identified in three different datasets with closely linked molecular markers. Out of these 35 loci, nine were resistance loci not previously identified, and five out of them are of particular interest as identified by multiple associations. The results of AM were validated in a durum wheat recombinant inbred line (RIL) population, in which a resistance locus was confirmed on the long arm of chromosome 6A. In addition, the information available for chromosomal positions of SNP markers was used to identify candidate genes for resistance based on map position and putative function.

## Materials and methods

### Plant material and genotyping

The stem rust reaction of a tetraploid wheat (*T. turgidum* L., 2*n* = 4*x* = 28; AABB genome) collection was assessed to identify resistance genes/QTLs via an association mapping approach. This collection consisted of 230 inbred lines classified into seven subspecies: ssp. *durum* (128), ssp. *turanicum* (20), ssp. *turgidum* (19), ssp. *polonicum* (20), ssp. *carthlicum* (12), ssp. *dicoccum* (19), and ssp. *dicoccoides* (12). Of the 128 durum wheat accessions, 96 represent the Italian durum wheat breeding programmes over the last 100 years of which seven are landraces or old durum wheat varieties (Cappelli, Aziziah, Russello, Timilia, Tangaron, Capeiti-8, and Grifoni). Laidò et al. (2013) provided a detailed list of the genotypes (number/name, year of release, country, pedigree) for each subspecies. The genetic diversity, population structure and linkage disequilibrium (LD) patterns of this collection of tetraploid wheats are fully described in Laidò et al. (2013, 2014). Each accession was genotyped with 26 simple sequence repeat (SSR) and 970 Diversity Arrays Technology (DArT) markers. Some durum wheat consensus maps are available, which contain information about the map positions of the microsatellite and DArT markers (Marone et al., 2012b; Maccaferri et al., 2015). We used the map by Marone et al. (2012b) as a reference in this study because it reports the chromosomal positions of 592 DArT markers that were used in the current AM panel for stem rust resistance. Furthermore, the maps by Marone et al. (2013) and Maccaferri et al. (2015) were also considered to identify EST-SSR, DArT and SNP markers located within the chromosome regions associated with stem rust resistance.

In addition, we evaluated the Cirillo (pedigree: Jucci/Polesine//Creso/Montanari)/Neodur (184-7/Valdur//Edmore) durum wheat population because the parents, which belong to the wheat tetraploid collection, exhibited a markedly different response to infection with race TTKSK. This population consists of 146 recombinant inbred lines (RILs). The genetic map for this population was comprised of 414 loci assigned to 30 linkage groups and spanning 1917 cM (Marone et al., 2012a; Russo et al., 2012).

### Phenotypic evaluation and statistical analysis

The stem rust evaluations were conducted in the Biosafety Level-3 Containment Facility on the St. Paul campus of the University of Minnesota (USA) during the winter of 2009–2010. Ten seeds of each RIL, the respective parents, accessions of the AM panel, and controls were sown into plastic pots (7.6 by 7.6 by 10.8 cm [length × width × height]) filled with a 50:50 mix of steam-sterilized native soil and Metro-Mix 200 (Sun Gro Horticulture, Quincy, MI; vermiculite, sphagnum peat moss, perlite, dolomitic limestone, and a wetting agent; Mamo et al., 2015). After planting, all pots were watered and fertilized with Osmocote controlled-release fertilizer 14-14-14 (Scott's Company, Marysville, OH) (1.4 g/pot) and Peters Dark Weather fertilizer 15-0-15 (Scott's Company) (~40 g/liter at 1/16 dilution). Plants were grown in the greenhouse at 19–22°C with a 14- to 16-h photoperiod supplemented by 400-W high-pressure sodium lamps emitting a minimum of 300 μmol photons m^−2^ s^−1^. Twelve-day-old seedlings (first leaf stage) were inoculated with freshly collected urediniospores of stem rust race TTKSK (isolate 04KEN156/04) suspended in a lightweight mineral oil (Soltrol 170; Phillips Petroleum, Bartlesville, OK) as a carrier. The concentration of inoculum used was 14 mg/0.7 ml oil applied at a rate of ~0.013 mg per plant (Steffenson et al., 2009). The oil carrier was allowed to evaporate before plants were moved into the mist chambers. Plants were then misted continuously for 30 min with ultrasonic humidifiers to establish an initial layer of moisture on the surfaces. Thereafter, plants were kept for 16–18 h in the dark at 20–22°C with periodic mistings from ultrasonic humidifiers. Following the infection period, plants were returned to the greenhouse under the same environmental conditions. Rust infection types (ITs) were scored on plants 12–14 days post-inoculation using the 0–4 scale described by Stakman et al. (1962), where IT = 0 represents the lowest incompatible (resistant) reaction and IT = 4 represents the fully compatible (susceptible) reaction. When IT = 0 (immune reaction) occurred, the test was repeated to exclude the possibility of disease escape. For AM analysis, categorical IT data were transformed to numeric data as follows: IT “0” was coded as 0.0; IT “0;” or “;” as 0.5; IT “1” as 2.0; IT “2” as 3.0; IT “3-”as 3.5; IT “3” as 4.0; and IT “3+” as 4.5; and IT “4” as 5.0 (Zhou et al., 2014).

The experiment was conducted in a completely randomized design and was repeated once over time. Any accessions exhibiting variable reactions across the replicates were repeated again in a third test. The susceptible wheat controls of McNair 701 (CItr 15288) and Line E (PI 357308) were included in each experiment to monitor the virulence of the race and the infection levels.

The data were analyzed using an ANOVA test, the homogeneity of phenotypic variance between replications was verified, and the means were separated by Fischer's protected least significant difference at *P* < 0.05 to test the difference across the tested genotypes. Genetic variance ($\sigma_{\text{G}}^{2}$) and broad-sense heritability (*H*) were estimated. All data were statistically analyzed using a statistical software package (Statistica, Statsoft Inc., Tulsa, OK, USA).

### Linkage and association mapping analysis

The phenotypic data obtained for the Cirillo × Neodur population clearly separated the RILs into the two phenotypic classes of resistance and susceptibility when compared to the phenotypes of the two parents. For this reason, the resistance was mapped as a single locus.

TASSEL software, version 3.0.115, was used to carry out general linear model (GLM) and mixed linear model (MLM) analyses for association mapping. For the GLM, genotypic data, phenotypic data and the *Q* matrix were integrated as covariates to correct for the effects of population substructure, which were determined with SSR markers for the whole collection (*K* = 2), the *durum* sub-sample (*K* = 3) and the Q2 group, which consists of wild and domesticated accessions (*K* = 2) (Laidò et al., 2014). In the MLM, the Kinship matrix (*K* matrix) was used in addition to the genotypic data, phenotypic data and *Q* matrix to correct for both population and family structure. The marker–phenotype association analysis was based on the polymorphisms present in 26 SSRs, and for DArT markers, at 958 loci in the whole collection, 845 loci in the *durum* sub-sample and 956 loci in the Q2 group. The loci considered for association mapping were characterized by a frequency of the rarest allele >0.1. The trimmed marker datasets were used to generate a marker similarity matrix containing all of the lines (*K* matrix) with the TASSEL software. TASSEL calculates the kinship as the proportion of alleles shared between each pair of lines. Once this matrix was calculated, the numbers were rescaled, so they were between 0 and 2 (Papa et al., 2007). The critical *P*-values for assessment of significance of the marker trait associations (MTAs) were calculated based on a false discovery rate (FDR) of 0.05 or 0.1 (Mosig et al., 2001), which is defined as the expected proportions of the true null hypotheses that are rejected. The algorithm described by Benjamini and Hockberg (1995) was used because it was shown to control FDR for independent test statistics, but also for some types of positive dependence (Benjamini and Yekutieli, 2001). FDR correction was applied on *P*-values obtained from GLM analysis. The same correction applied to *P*-values from MLM analysis did not produce significant results. In order to verify the robustness of the results, a model was run with 10 genes whose haplotypes were designed for them to explain each around 5% variance in the MLM model. The 10 genes were analyzed in the whole collection dataset as it was characterized by an heritability very close to 0.8, and with minor allele frequency of 0.1. Results of the simulation are reported in Figure S1.

Two independent approaches were used to locate unmapped MTAs for stem rust resistance identified with the three datasets employed in this study. The first was based on the calculation of *linkage disequilibrium* (LD) among the unmapped MTAs and the 592 DArT markers located on the durum wheat consensus map (Marone et al., 2012b). Unmapped markers were assigned the map position of mapped markers with which they were in strong or complete LD. The second approach was based on the projection of chromosome regions harboring unmapped MTAs that were previously published on the durum wheat consensus map (Marone et al., 2013) using the Biomercator software (Goffinet and Gerber, 2000).

Overall, we obtained three classes of significance: (i) MTAs with *P* < 0.05 in MLM and FDR of 0.05 in GLM; (ii) MTAs with *P* < 0.05 in MLM and FDR of 0.1 in GLM; and (iii) MTAs with *P* < 0.05 in both MLM and GLM, or MLM. We decided to report also the MTAs in the third significance class, as many of them are confirmed by linkage mapping or literature data and therefore we think that excluding these MTAs could determine a large loss of power in our analysis (see Section Results for details). We considered as associated markers, all of the significant MTAs identified in both models (GLM and MLM). We also reported those associated only in the MLM model but located in chromosomal regions harboring *Sr* genes. Moreover, all of the MTAs mapped on the durum wheat consensus map (Marone et al., 2012b) within a short map interval (15 cM or less) were grouped into a single QTL. To compare our results with those obtained in other studies, we considered the most recently published information on genes and QTL mapping for the stem rust resistance.

## Results

### Evaluation of the tetraploid wheat collection for resistance to stem rust

In all of the seedling tests, the susceptible controls of McNair 701 and Line E were heavily infected and exhibited the expected compatible ITs ranging from 3 to 4 to race TTKSK. The high levels of infection achieved in each experiment allowed for the reliable scoring of ITs on all accessions.

A high level of variability was observed in response to stem rust race TTKSK in the tetraploid wheat collection (Figure S2), thereby allowing for the identification of resistance loci in both the domesticated subgroups and the gene pool of cultivated durum wheat cultivars. Over one-fifth (46–20.5%) of the 230 tetraploid wheat accessions were rated as resistant (ITs 0; 1; or 2) to race TTKSK (Table 1 and Table S1). In particular, all ssp. *dicoccoides* accessions were susceptible, and only one accession of ssp. *turgidum* (PI 191104) was resistant with IT of 2 (Table 1 and Table S1). For the other subspecies, two accessions of ssp. *turanicum* (PI 184526 and PI 191599), two of ssp. *polonicum* (PI 366117 and PI 387479), two of ssp. *dicoccum* (ISC Foggia 171 and MG 5323), and three of ssp. *carthlicum* (PI 115816, PI 572849, and PI 585018) were rated as resistant. Within ssp. *durum*, 24, 25%, of the 96 Italian durum wheat varieties were rated as resistant, whereas just 12 accessions from other countries were resistant (Table 1 and Table S1).

**Table 1:** **Statistical estimations for each dataset and summary of resistant and susceptible accessions for each subspecies included in the whole collection, in the ***durum*** subsample and in the Q2 group**.

**Dataset**	**Tetraploid wheat classification (AABB) MacKey** ***T. turgidum*** **ssp**.											
	**No. of accessions**		***Durum***	***Turanicum***	***Polonicum***	***Turgidum***	***Carthlicum***	***Dicoccum***	***Dicoccoides***	**No. of accessions**	***H*^2^**	**LSD (0.05)**
Whole collection	230	S	91	17	18	17	9	15	12	179	0.93	0.51
		R	36	2	2	1	3	3	0	47		
		MS	1	1	–	1	–	1	–	4		
*Durum* subsample	128	S	91	–	–	–	–	–	–	91	0.93	0.5
		R	36	–	–	–	–	–	–	36		
		MS	1	–	–	–	–	–	–	1		
Q2 group (K2–26 SSR markers)	101	S	6	14	14	17	9	15	12	87	0.74	1.05
		R	–	2	2	1	3	3	–	11		
		MS	–	1	–	1	–	1	–	3		

*H ^2^, heritability; LSD, least significant difference; S, susceptible; R, resistant; MS, moderately susceptible*.

The analysis of variance for stem rust reaction showed highly significant differences (*P* < 0.0001) among accessions included in the whole collection, the *durum* sub-sample and Q2 group. The statistical parameters of stem rust response are given in Table 1, and the heritability ranged from 0.74 in the Q2 group to 0.93 in the whole collection and in the durum sub-sample. This result indicates the robustness of the data and the low error rate.

### Association mapping

**Figure 1:**
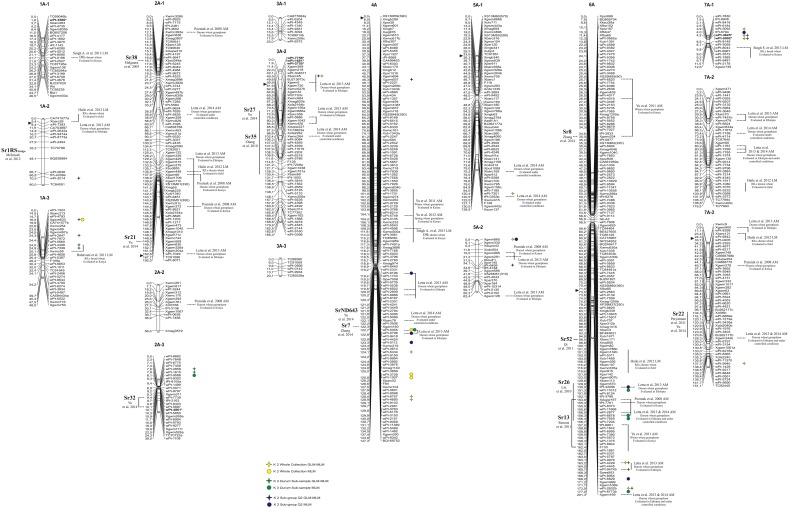
**Durum wheat consensus linkage map (genome A—reported in Marone et al., 2012b) with the genetic positions of markers significantly associated with resistance to stem rust race TTKSK at seedling stage in the whole collection (yellow), in the ***durum*** subsample (green), and in the Q2 group (blue). Right:** circles, MTAs identified with the MLM; stars, MTAs identified in both GLM and MLM; the markers in bold and with an asterisk indicate MTAs that have been projected on the durum wheat consensus map.

**Figure 2:**
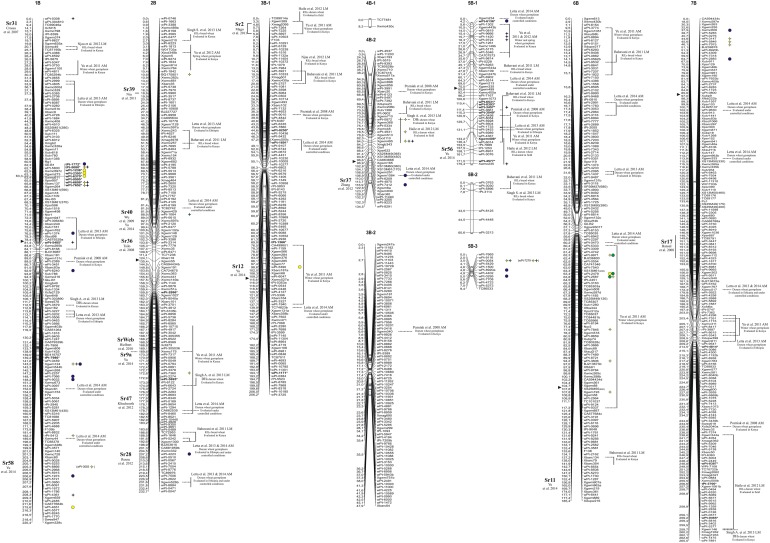
**Durum wheat consensus linkage map (genome B—reported in Marone et al., 2012b) with the genetic positions of markers significantly associated with resistance to stem rust race TTKSK at seedling stage in the whole collection (yellow), in the ***durum*** subsample (green) and in the Q2 group (blue)**. **Right**: circles, MTAs identified with the MLM; stars, MTAs identified in both GLM and MLM; the markers in bold and with an asterisk indicate MTAs that have been projected on the durum wheat consensus map.

Taking into account the MTAs detected in both models and those found associated in the MLM located in chromosomal regions harboring previously described *Sr* genes, 35 QTL were identified for seedling response to race TTKSK across the genome (Table 2), of which 12 were of the highest significance level. These QTL were found on all chromosomes and were represented by either sets of closely linked markers (17, Table 3), or single markers (18, Table 4). The QTL identified by multiple significant associations were defined by sets of closely linked SSR and DArT markers, significantly associated with the phenotype and located within chromosomal regions of 15 cM or less, with the same directional effect. The marker with the most significant association was considered as the QTL-tagging marker. The results of the genome scan for the stem rust response are summarized in Tables 3, 4, and in Figures 1, 2, along with a summary of the most recently published information on *Sr* genes and QTL conferring resistance to races in the Ug99 lineage. Coincidence between our association mapping results in tetraploid wheat and those reported in previous studies was observed in several cases (see Figures 1, 2 and Section Discussion). Some of the identified loci for resistance were of particular interest, for different reasons. A number of QTL were identified as multiple associations and were significant in more than one independent dataset. The QTL tagged by the marker Xgwm495 (group 4B-2) was highly significant in the whole collection and was identified also in the other two datasets used in the present study, albeit at a lower significance level (*R*^2^ = 3–6% and 4–5%). A similar behavior was observed for the marker Xgwm124 (chromosome 1B). Other QTL were common to two datasets: the one tagged by the marker tPt-5080 (chromosome 1B) was common to the whole collection and the Q2 group (*R*^2^ = 2–7%), while those with peak markers Xgwm633 (group 1A-3), wPt-0054 (group 5B-3), wPt-4229 (chromosome 6A), wPt-9971 (chromosome 6B), and wt-0002 (group 7A-1) were significant in both the whole collection and the *durum* subsample (*R*^2^ = 2–5%). As for the portion of explained phenotypic variability, the QTL with the highest *R*^2^ were positioned on chromosomes 1B (wPt-4361, 10%), 1A-2 (wPt-2014, 9%), and 4A (Xgwm937, 9%). All of these MTAs were identified in the Q2 group.

**Table 2:** **Comparison of the two models used for calculation of the associations between the mapped and unmapped markers, and the trait, for the whole collection, the ***durum*** sub-sample and the Q2 group**.

**Dataset**	**No. of accessions**	**N. MTAs with MLM**	**N. MTAs common to GLM and MLM (%)**	**N. MTAs identified with MLM and coincident with known *Sr* genes**
Whole collection	230	82	51 (24.9%)	5
*Durum* subsample	128	38	24 (21.1%)	3
Q2 group	101	119	22 (10.1%)	8

*GLM, general linear model (Q matrix); MLM, mixed linear model (Q matrix + kinship matrix); sign., significant with P < 0.05*.

**Table 3:** **QTL regions represented by multiple MTAs for seedling resistance to stem rust race TTKSK in the whole collection (bold), in the ***durum*** subsample (italics) and in the Q2 group (underlined)**.

**Marker**	**Chr**.	**Position (cM)**	**Whole Collection**			***Durum Subsample***			**Q2 group**			**Other MTAs in the region**	**Range *R*^2^(%)**	**Chr. Interval**
			***P*-value (Q) GLM (FDR threshold)**	***P*-value (Q + K) MLM**	***R*^2^ MLM (%)**	***P*-value (Q) GLM (FDR threshold)**	***P*-value (Q + K) MLM**	***R*^2^ MLM (%)**	***P*-value (Q) GLM (FDR threshold)**	***P*-value (Q + K) MLM**	***R*^2^ MLM (%)**			
tPt-5080^*^	1B	63.3	2.8E-04 (0.05)	1.9E-02	2	–	–	–	1.6E-06 (0.05)	1.0E-02	7	wPt-7652^*^, wP-t6608^*^, tPt-1772^*^, wPt-1238^*^, wPt-2395^*^, wPt-6370^*^	2–7	0.0
wPt-4680	4A	128.9	9.4E-05 (0.05)	3.8E-02	2	–	–	–	–	–	–	wPt-9196, wPt-0798, wPt-5112, **wPt-5434, wPt-1007**, **wPt-3729**, **wPt-6757**, ***wPt-5055***	2–8	9.7
Xgwm495	4B-2	84.0	1.0E-03 (0.05)	2.9E-03	4	8.4E-03 (–)	2.4E-02	4	2.0E-03 (–)	3.6E-02	5	**wPt-0872**, **wPt-4931**	2	9.6
wPt-9724	5B-1	16.3	–	–	–	–	–	–	5.7E-04 (0.05)	1.7E-02	6	wPt-9666, wPt-1420, wPt-6136^*^	5-8	12.1
wPt-0054	5B-3	3.5	3.0E-03 (0.05)	1.7E-02	3	3.2E-03 (–)	3.4E-02	4	–	–	–	wPt-8920, wPt-0295	5-7	1.5
wPt-4229	6A	163.2	2.6E-04 (0.05)	1.4E-02	3	2.1E-03 (–)	1.4E-02	5	–	–	–	*wPt-9474*, wPt-6829, ***wPt-2632**, wPt-8773*	3-7	13.8
wPt-9971	6B	62.3	9.6E-04 (0.05)	4.0E-03	4	–	4.7E-02	3	–	–	–	***wPt-2479, wPt-2587***	2–4	1.5
wPt-3606	6B	94.2	2.3E-03 (0.05)	2.7E-03	4	–	–	–	–	–	–	**Xgwm193**	2	13.6
Xgwm124	1B	143.2	6.3E-03 (0.1)	2.3E-02	2	7.5E-03 (–)	6.5E-03	6	–	1.7E-02	6	wPt-7066, wPt-9032	6	5.0
wPt-4361	1B	208.3	–	–	–	–	–	–	2.2E-03 (0.1)	2.6E-02	10	wPt-1973, **wPt-4651**	2–7	9.0
wPt-0002^*^	7A-1	4.2	6.3E-03 (0.1)	2.5E-02	2	2.3E-03 (–)	2.2E-02	5	–	–	–	wPt-4625^*^, tPt-6794, **wPt-5092**	2–6	0.3
wPt-7975	7B	46.1	1.4E-02 (0.1)	5.1E-03	4	–	–	–	–	–	–	**wPt-3147, wPt-5846**, Xwmc606	3–5	8.6
Xgwm633	1A-3	16.0	–	1.5E-02	3	2.2E-02 (–)	2.6E-02	4	–	–	–	*wPt-2847, wPt-4408, wPt-7339*	4–5	8.9
wPt-5485^*^	1B	81.4	–	–	–	–	–	–	1.8E-02 (–)	1.3E-02	7	wPt-1374	5	4.9
wPt-2858	2A-3	7.6	–	–	–	4.9E-02 (–)	3.7E-02	3	–	–	–	*wPt-9586, wPt-1615*	3–4	0.5
Xgwm1084	4B-2	106.9	–	–	–	–	–	–	–	4.5E-04	4	wPt-7412	4	10.0
wPt-11612	6A	151.3	–	–	–	–	3.2E-02	4	–	–	–	*wPt-7655, wPt-6678*	4–5	5.1

Asterisks (^*^) indicate the DArT markers projected on the durum wheat consensus map. Chr, chromosome.

**Table 4:** **Single MTAs for seedling resistance to stem rust race TTKSK in the whole collection and/or in the Q2 group**.

**Marker**	**Chr**.	**Position (cM)**	**Whole Collection**			***Durum*** **Subsample**			**Q2 group**			**Literature**
			***P*-value (Q) GLM (FDR threshold)**	***P*-value (Q + K) MLM**	***R*^2^ MLM (%)**	***P*-value (Q) GLM (FDR threshold)**	***P*-value (Q + K) MLM**	***R*^2^ MLM (%)**	***P*-value (Q) GLM (FDR threshold)**	***P*-value (Q + K) MLM**	***R*^2^ MLM (%)**	
rPt-5412	1B	182.3	1.7E-03 (0.05)	6.7E-03	3	–	–	–	–	–	–	
BQ170801	2B	10.5	4.7E-04 (0.05)	2.3E-02	2	–	–	–	–	–	–	
wPt-7360	2B	172.8	4.8E-05 (0.05)	3.6E-02	2	–	–	–	–	–	–	*Sr47*
Barc45	3A-2	49.5	5.1E-04 (0.05)	1.7E-02	3	–	–	–	2.9E-03 (0.1)	2.6E-02	5	
wPt-2014	1A-2	70.5	–	–	–	–	–	–	5.2E-03 (0.1)	3.9E-03	9	
Xgwm937	4A	46.3	–	–	–	–	–	–	3.2E-03 (0.1)	4.7E-03	9	
wPt-7201	5A-1	120.4	4.8E-03 (0.1)	3.5E-02	2	–	–	–	–	–	–	
Xgwm865	5A-2	0.0	1.4E-02 (0.1)	4.9E-02	2	–	–	–	–	3.9E-02	4	
wPt-7846	6B	77.5	3.4E-03 (0.1)	2.7E-02	2	–	–	–	–	–	–	
wPt-5940	7A-3	137.3	6.5E-03 (0.1)	1.6E-02	3	–	–	–	–	–	–	
wPt-0308	1B	0.0	–	–	–	–	–	–	7.1E-03 (–)	1.0E-02	7	
wPt-1064	2B	88.9	–	–	–	1.8E-02 (–)	4.9E-02	3	–	–	–	
wPt-4223	2B	207.0	–	–	–	–	–	–	–	1.4E-02	6	*Sr28*
wPt-5390	3B-1	98.8	–	4.8E-02	2	–	–	–	–	–	–	
tPt-6495	5A-2	34.6	–	–	–	–	–	–	2.7E-02 (–)	4.8E-02	4	
wPt-3457^*^	5B-1	110.4	–	–	–	–	–	–	2.0E-02 (–)	2.2E-02	5	
Xwmc235	5B-1	175.4	–	–	–	–	–	–	6.1E-03 (–)	9.2E-03	7	
tPt-4209b	6A	130.0	–	–	–	–	–	–	–	1.4E-02	6	*Sr26*

Asterisks (^*^) indicate the DArT markers projected on the durum wheat consensus map. Chr, chromosome.

In most cases, alleles associated with resistance to stem rust were common in the collection, with allele frequency around 0.5. However, in some cases the resistance was associated with rare alleles in a frequency range of 0.1–0.2. The presence allele of the peak marker wPt-0054 (group 5B-3) showed an effect of −0.33 (in increasing score and therefore in increasing resistance) with a frequency of 0.19 in the whole collection. The same behavior was observed for Xgwm865 (group 5A-2), which showed an effect of −0.49 with a frequency of 0.1 in the whole collection (data not shown).

Finally, most of the regions identified as harboring resistance in the present study were coincident with QTL previously published (see Figures 1, 2 and Section Discussion). As an example, the MTAs wPt-7360, wPt-1646, and wPt-4223 were located on chromosome arm 2BL, where different *Sr* genes have been previously reported (Hiebert et al., 2010; Klindworth et al., 2012; Rouse et al., 2012; Yu et al., 2014) (Figure 2). For this reason, we were particularly interested in QTL mapping to putative novel locations, such as those tagged by the marker wPt-5080 (chromosome 1B) in the whole collection and the Q2 group, and wPt-0054 (group 5B-3) in the whole collection and the *durum* subsample. To the best of our knowledge, a new region for a stem rust resistance QTL was tagged by the marker wPt-2858 on group 2A-3 in the *durum* subsample; in this case the resistance allele had an effect of –0.44 with a frequency of 0.13.

### Identification of putative candidate genes for resistance to stem rust

In order to identify candidate genes for stem rust resistance, we inspected the putative function of gene sequences corresponding to markers in the regions of identified QTL, as reported by Marone et al. (2013). Moreover, we also considered the putative function of genes corresponding to the SNP markers (Wang et al., 2014) located on the durum wheat consensus map reported by Maccaferri et al. (2015) that were near to the MTAs identified in this study. As reported in Figure S3, many gene families were identified, although some functional categories were more frequently represented than others. In particular, a high proportion of genes corresponded to sequences related to disease resistance in plants, such as NBS-LRR resistance genes (15) and protein kinases (171) many of which contained an LRR domain (20). Other classes were represented by E3 ubiquitin ligases (most of which contain an F-box domain), and various kinds of membrane transporters and proteases. In Table 5, all of the genes putatively involved in response to pathogens which have been mapped near the MTAs are reported. It is important to emphasize that due to the LD extent present in the germplasm evaluated, the presence of disease-response genes can be observed by chance. Nevertheless, in some cases this presence may be of interest, as the MTA itself corresponds to a disease resistance gene. An example is the DArT marker wPt-0308 (*R*^2^ = 7%) which was significantly associated in the Q2 group, and corresponds to a gene encoding a vacuolar sorting protein 39 involved in the *Reaction to P. graminis 1* (*Rpg1*)-mediated stem rust resistance in barley (Nirmala et al., 2011) (Table 5). The QTL consisting of three MTAs (QTL-tagging marker wPt-11612) in an interval of 5.1 cM of the long arm of chromosome 6A was identified in the ssp. *durum* subsample with *R*^2^-values from 4 to 5% (Table 3). In this chromosome interval, Simons et al. (2011) mapped the stem rust resistance gene *Sr13* based on the common marker Xdupw167 (Figure 1). Moreover, the MTA wPt-7655 corresponds to a gene encoding a 1,3-beta-glucan synthase component (Table 5). The QTL tagged by marker wPt-4680 on chromosome 4A was represented by a set of nine adjacent MTAs, located on the durum wheat consensus map in an interval of 9.7 cM, with *R*^2^-values from 2 to 8% (Table 3 and Figure 1). Several candidate genes corresponding to NBS-LRR proteins were reported in this interval by Marone et al. (2013) and Maccaferri et al. (2015) (Table 5).

**Table 5:** **Candidate genes mapped in chromosome regions identified as associated with seedling resistance stem rust race TTKSK**.

**Durum Wheat Consensus Map (Marone et al., 2012b)**				**DArT markers in the QTL region based on the map by Marone et al., 2013**	**SNP markers in the QTL region based on the map by Maccaferri et al., 2015**
**Chr**	**QTL**	**Position**	**Putative function**		
1A-3	Xgwm633–wPt-7339	16.0–24.9	–	wPt-0432 (14.9 cM from wPt-2847) NBS-LRR	IWA7924 (5.5 cM from wPt-7339) leucine-rich repeat protein kinase putative expressed subfamily LRR-XII
1B	wPt-0308	0.0	Vacuolar sorting protein 39	–	IWB44529, IWB57219 (2.2 and 2.5 cM from wPt-0308) NBS-LRR
	wPt-5485	81.4	–	–	IWB19579 (0.6 cM from wPt-5485) CAMK_KIN_like.18—CAMK includes calcium-calmodulin dependent protein kinases
	Xgwm124–wPt-7066	143.2–148.2	NBS-LRR (wPt-9032)	wPt-1403 (2.9 cM from Xgwm124) Serine/Threonine protein kinases	IWB50556 (0.8 cM from wPt-7066) NBS-LRR
	wPt-4361–wPt-4651	206.6–215.6	–	–	IWB57627, IWB55003 (0.3 and 10.7 cM from wPt-4361) NBS-LRR
2A-3	wPt-2858–wPt-9586	7.6-8.1	–	–	IWB38972, IWB51340, IWB70745 (3.8, 4.7, and 4.7 cM from wPt-2858) NBS-LRR
2B	BQ170801	10.5	–	wPt-4026, wPt-8970 (10.0 and 6.8 cM from BQ170801) NBS-LRR	IWB56084, IWB57438 (5.6 and 5.6 cM from BQ170801) leucine-rich repeat protein kinase putative expressed subfamily LRR-XII
	wPt-1064	88.9	–	wPt-2120b (1.1 cM from wPt-1064) NBS-LRR	IWB36128, IWB31001 (0.0 and 0.3 cM from wPt-1064) CAMK_KIN_like5—CAMK includes calcium-calmodulin dependent protein kinases
	wPt-7360	172.8	–	wPt-0189 (3.0 cM from wPt-7360) NBS-LRR	IWA7630 (1.4 cM from wPt-7360) protein kinase family protein putative expressed subfamily RLCK-XII
	wPt-4223	207.0	–	Xcdo244 (1.7 cM from wPt-4223) NBS-LRR; wPt-9257 (0.4 cM from wPt-4223) LRR/KIN	IWB21522 (5.4 cM from wPt-4223) protein kinase family protein putative expressed subfamily SD-1c
3A-2	Barc45	49.5	–	wPt-2698 (0.3 cM from Barc45) PHD zinc finger protein-like; BJ213673c (0.0 cM from Barc45) CTD-phosphatase-like protein	IWB24651 (6.7 cM from Barc45) protein kinase family protein putative expressed subfamily SD-2b
3B-1	wPt-5390	98.8	–	wPt-0644 (0.7 cM from wPt-5390) Leucine Rich repeats (2 copies)	IWB47274 (4.9 cM from wPt-5390) protein kinase family protein putative expressed subfamily Raf
					IWB32491 (2.7 cM from wPt-5390) leucine-rich repeat protein kinase putative expressed subfamily LRR-XIIIa
	wPt-5055–wPt-4680	119.2–128.9	NBS-LRR (wPt-3729)	wPt-4487a, wPt-0833a, wPt-2951 (0.8, 2.3, and 5.1 cM from wPt-4680) NBS-LRR; wPt-0992a (2.4 cM from wPt-4680) NBS2-RDG2A	IWB26676, IWB61552 (0.0 and 0.5 cM from wPt-1007) NBS-LRR
4A	Xgwm937	46.3	–	wPt-6303, wPt-4660 (8.1 and 10.0 cM from Xgwm937) NBS-LRR	–
4B-2	Xgwm495–wPt-4931	78.5–84.0	–	Xbcd110 (0.8 cM from Xgwm495) filament-like plant protein 4-like	IWB29759 (0.7 cM from wPt-4931) CAMK_KIN_like.11—CAMK includes calcium-calmodulin dependent protein kinases
	Xgwm1084–wPt-7412	106.9–116.9	–	–	IWB47175 (4.6 cM from Xgwm1084) CAMK_CAMK_like.18—CAMK includes calcium-calmodulin dependent protein kinases
5A-1	wPt-7201	120.4	–	–	IWA3827 (3.2 cM from wPt-7201) protein kinase binding
5A-2	tPt-6495	34.6	–	–	IWB56756 (5.7 cM from tPt-6495) CAMK_CAMK_like_ULKh_APGy.2—CAMK includes calcium-calmodulin depedent protein kinases
5B-1	wPt-6136^*^–wPt-9724	4.2–16.3	Protein kinase domain (wPt-6136^*^)	wPt-9800b (20.7 cM from wPt-9724) NBS-LRR; wPt-0819 (3.8 cM from wPt-9724) LRRs, RI-like subfamily; wPt-8604 (0.8 cM from wPt-9724) LRR receptor-like PK	–
	Xwmc235	175.4	–	–	IWA6053 (4.8 cM from Xwmc235) CAMK_CAMK_like.1—CAMK includes calcium-calmodulin dependent protein kinases expressed subfamily
5B-3	wPt-0054–wPt-0295	3.5–5.0	–	wPt-5168 (5.6 cM from wPt-0054) leucine-rich repeat receptor-like protein kinase	IWB28597 (1.3 cM from wPt-0054) protein kinase family protein putative expressed subfamily Raf
6A	tPt-4209	130.0	–	wPt-8331 (7.8 cM from tPt-4209) NBS-LRR	IWB26292 (0.3 cM from tPt-4209) protein kinase family protein putative expressed subfamily Raf
	wPt-11612–wPt-6678	151.3–156.4	1,3-beta-glucan synthase component (wPt-7655)	wPt-3191a (6.3 cM from wPt-11612) NBS-LRR	–
	wPt-4229–wPt-8773	163.2–177.0	–	–	IWB22871 (1.6 cM from wPt-4229) protein kinase family protein putative expressed subfamily SD-2b
					IWA7572 (7.4 cM from wPt-4229) leucine-rich repeat protein kinase putative expressed subfamily LRR-XII
6B	wPt-9971–wPt-2587	62.3–63.8	–	wPt-7540, wPt-8153 (0.5 and 3.3 cM from wPt-9971) NBS-LRR	IWB48366 (0.4 cM from wPt-9971) NBS-LRR
	wPt-7846	77.5	F-box domain (wPt-7846)	–	IWB55473 (7.2 cM from wPt-7846) leucine-rich repeat protein kinase putative expressed subfamily LRR-XV
	wPt-3606–Xgwm193	94.2–107.8	–	wPt-8721 (2.3 cM from wPt-3606) putative mitogen-activated protein kinase 17-3	IWB25027 (0.0 cM from Xgwm193) leucine-rich repeat protein kinase putative expressed subfamily LRR-XII
7A-3	wPt-0002^*^–wPt-5092	3.9–4.2	–	–	IWB42938 (5.7 cM from wPt-0002) NBS-LRR
	wPt-5940	137.3	–	TC92445 (11.1 cM from wPt-5940) pathogenesis-related protein 1-15	–
7B	Xwmc606–wPt-7975	37.5–46.1	–	wPt-9800c (12.8 cM from wPt-7975) NBS-LRR	–

Asterisks (^*^) indicate the DArT markers projected on the durum wheat consensus map.

### Linkage mapping

**Figure 3:**
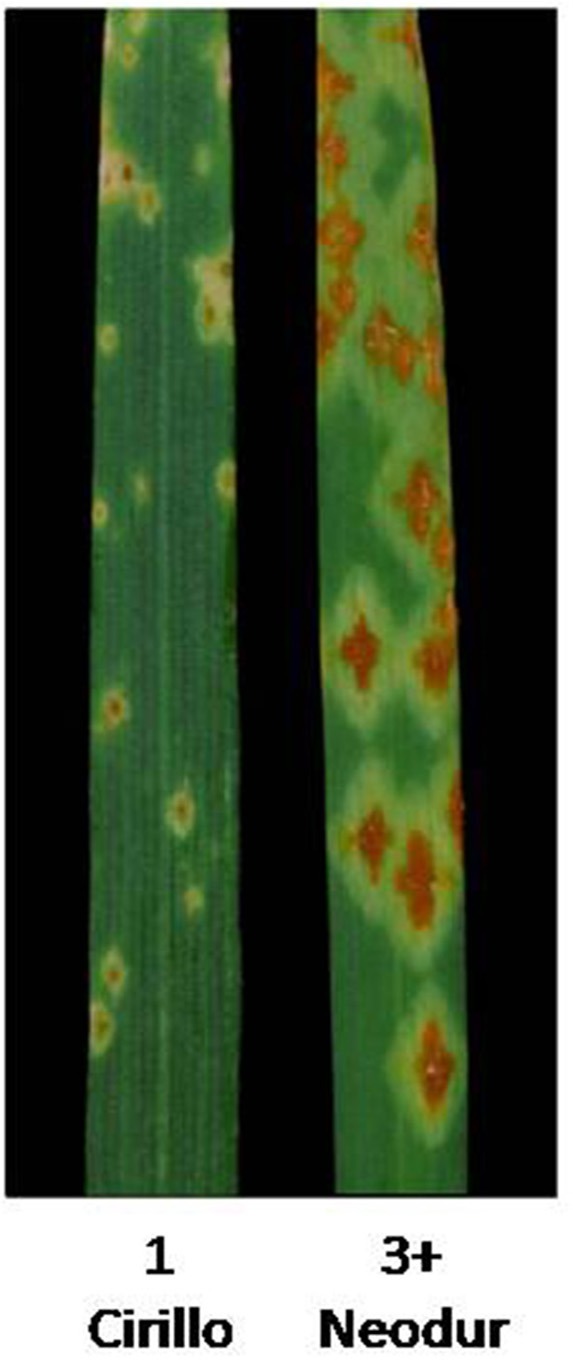
**Stem rust Infection types (IT) exhibited by mapping parents “Cirillo” (resistant, ***IT*** = 1) and “Neodur” (susceptible, ***IT*** = 3+) using the scoring system of Stakman et al. (1962)**.

In order to validate, at least in part, the results of AM analysis, we used a linkage mapping approach based on the output of the phenotypic evaluation of the tetraploid wheat collection. A clear difference was observed for the durum wheat cultivars Cirillo, exhibiting IT 1, and Neodur IT 3+ (Table S1, Figure 3). As these genotypes are parents of a RIL population, the evaluation of ITs was performed on the entire segregating population. All of the RILs were easily classified as resistant or susceptible because they exhibited reactions very similar to that of the parents. Furthermore, the ratio of phenotypic scores in response to race TTKSK confirmed the presence of a single gene segregating in the RIL population (*X*^2^ = 0.68, *P* < 0.05). Therefore, the stem rust resistance of Cirillo was mapped as a single Mendelian gene. The segregation data were integrated into the Cirillo × Neodur linkage map, spanning 1917 cM and comprising 202 PCR-based and 212 DArT markers (Marone et al., 2012a). The resistance gene effective against race TTKSK was positioned to chromosome arm 6AL, between the markers wPt-8124 and Xdupw167, very near to marker wPt-11612, which was identified as a MTA in the *durum* sub-group in the AM analysis (Figure 1). Moreover, these molecular markers were positioned in an interval of 1.9 cM on the consensus map by Marone et al. (2012b), a region where Simons et al. (2011) mapped the resistance gene *Sr13* in the UC1113 × Kofa segregating population. The effect of the peak marker in the AM panel was further investigated. The resistant parent Cirillo showed the allele “1” (presence) at the locus wPt-11612, while the susceptible parent Neodur showed the allele “0” (absence). As shown in the allele effect plot reported in Figure S4, the effect of allele “1” in decreasing susceptibility is 0.6, in phase with the Cirillo resistance allele mapped in the segregating population.

## Discussion

A better understanding of the genetic basis underlying the response to African stem rust races will help in enhancing the disease resistance of durum wheat. To this end, association mapping is a useful approach as indicated by the growing interest in its application to identify disease resistance genes/QTL in a wide range of crops (Hall et al., 2010; Letta et al., 2013). In the present investigation, we have conducted a genome-wide association study to identify chromosome regions involved in resistance to race TTKSK in the Ug99 lineage of African stem rust races. Using a structured panel of tetraploid wheat accessions (230 inbred lines), including a large set of durum wheat varieties and a representative sample of *T. turgidum* evolutionary lineages, including wild and domesticated accessions, we performed an association analysis on three samples (whole collection, *durum* subsample and Q2 group) with different LD levels and structures (Laidò et al., 2014) with the possibility of cross validation of results and for identification of new resistance loci.

Previous genetic studies on the resistance of wheat to races in the Ug99 lineage under field conditions have suggested that resistance is likely to be under oligenic or polygenic additive control, resulting from the cumulative effect of beneficial alleles from multiple loci (major and minor) of variable effect. This hypothesis was supported by different genetic mapping approaches, conducted using segregating populations (Haile et al., 2012) and germplasm collections (Letta et al., 2013), in which stem rust resistance was associated with several genomic regions, each contributing a small fraction of the phenotypic variability under field conditions. In the present study, the reaction to race TTKSK, the most prominent race in the Ug99 lineage, was evaluated at the seedling stage, and 35 QTL, at various significance levels, were identified for resistance. Each QTL contributed a small fraction (<10%) of the phenotypic variation explaining the trait. The QTL identified in the present investigation were located on all of the chromosomes (Tables 3, 4). According to the different LD levels detected in the three datasets, the durum subsample, characterized by extensive LD among markers, showed QTL regions tagged by multiple marker-trait associations while in the Q2 group, with faster LD decay rate, associations were mostly based on single significant markers.

The structured panel of tetraploid wheat accessions evaluated in the present study encompasses a large portion of the genetic variation present in the gene pools and therefore is a good resource for identifying new stem rust resistance genes. Resistance sources could be identified among domesticated accessions, but also within the gene pool of cultivated durum wheat. In particular, out of the 36 genotypes of ssp. *durum* that were scored as resistant with IT = 2, nine exhibited very low ITs between 0 and 1, and five are Italian durum wheat varieties (Athena, Provenzal, Tiziana, Simeto, Zenit). Having effective sources of resistance in elite cultivars represents a huge advantage in breeding as the resistance determinants are already present in an adapted genetic background with respect to agronomic and quality traits. In the alternative case, the transfer of a resistance locus from a wild or domesticated accession runs the risk of introducing linked deleterious alleles (i.e., linkage drag) in the recurrent parent. Nevertheless, a large number of effective resistance genes are needed by breeders to counter the evolution of virulence determinants in the pathogen.

The comparison of our results with those previously reported in the literature on mapping resistance loci to Ug99 lineage stem rust races corroborated many of the identified MTAs from this study and clearly indicates that our approach was successful. Key to this success was the high heritability observed for the targeted trait (from 74% in the Q2 group to 93% in the whole collection) since association mapping is strongly influenced by heritability and the quality of phenotypic data (Rafalski, 2010; Pasam et al., 2012). The MTAs identified in the present study are reported as positioned on the durum consensus map by Marone et al. (2012b) in Figures 1, 2. Based on common molecular markers, resistance loci previously mapped in the same regions are also reported. In many cases, we found a correspondence between the origin of the resistance locus previously identified and the panel in which the MTA was identified. Chromosome 1BS carries *Sr14*, which is ineffective against race TTKSK (Jin et al., 2007). *Sr14* can be traced back to *Triticum dicoccum* Schrank accessions such as Khapli emmer (Heermann and Stoa, 1956), considered as one of the few founders of modern durum wheat germplasm (Autrique et al., 1996). Accordingly, we found the MTA wPt-0308 only in the Q2 group. Moreover, since the *Sr14* gene is ineffective against the race TTKSK, the resistance locus identified in the present study could represent a different allele at the same locus, or a different closely linked resistance gene. Allelism tests should be conducted in the future to resolve this question.

The two QTL detected by markers tPt-4209 and wPt-11612 on the long arm of chromosome 6A were mapped to chromosome regions previously reported to carry the resistance genes *Sr26* and *Sr13*, respectively (Simons et al., 2011). Both genes are effective against the primary race components of the Ug99 lineage, namely TTKSK (Ug99), TTKST, and TTTSK (Singh et al., 2006). *Sr13* was identified in several *T. turgidum* ssp. *durum* cultivars, and Simons et al. (2011) reported its map position at a distance of 2.8 and 0.6 cM from the marker Xdupw167 in the UC1113 × Kofa and Kronos × Rusty populations, respectively. Letta et al. (2013, 2014) identified a QTL region for resistance to a number of stem rust races corresponding to *Sr13* in a durum wheat panel via association mapping. In our investigation, three MTAs (wPt-11612, wPt-6678, and wPt-7655) were mapped in an interval of 2.6 cM, and marker Xdupw167 was located in the same interval (Figure 1). These MTAs are located in the same region identified by Letta et al. (2013, 2014). *Sr13* was previously identified only in durum wheat cultivars. Accordingly, we found MTAs in the same chromosome region only in the durum subsample in our analysis. Moreover, this locus was mapped as the resistance determinant in the durum wheat cultivar Cirillo by linkage mapping in the Cirillo × Neodur population.

In addition to confirming a number of previously reported MTAs, we also identified numerous resistance loci that appear to map in unique chromosomal regions (Figures 1, 2). In particular, stem rust resistance QTL identified on chromosomes 1BL, 2AL, 5BL, 7BS (QTL-tagging markers wPt-4361, wPt-2858, wPt-0054, and wPt-7975, respectively), and the MTAs detected on chromosomes 1AL, 1BS, 4AL, and 7AL (wPt-2014, wPt-0308, Xgwm937, and wPt-5940, respectively) were not reported elsewhere to the best of our knowledge. The fact that they were associated with resistance in both models (GLM and MLM), and in some cases in two different panels, confirms the robustness of the results of this association mapping analysis. Moreover, in some cases, even if some QTL mapped to regions harboring previously characterized *Sr* genes, the MTAs identified in the present investigation could represent new alleles/genes for resistance.

The QTL tagged by the marker wPt-0308 (1BS) was mapped to a region known to carry *Sr31* (Yu et al., 2014). Its presence in durum wheat is unlikely because this gene has been transferred from *Secale cereale* to bread wheat (Olson et al., 2013). Consequently, the MTA wPt-0308, detected in the Q2 group, appears to tag a new resistance locus. Allelism tests and further phenotypic analyses with a suite of different stem rust races will be needed to resolve this question.

## Conclusions

Our data show that seedling resistance to race TTKSK of the Ug99 lineage in tetraploid wheat is controlled by a large number of QTL, and among these, several are located in chromosomal regions for which no previously designated loci have been mapped.

By genotyping collections of accessions and cultivars with a large number of markers, association studies provide the means to improve the genetic characterization of germplasm. Markers associated with genomic regions that control the resistance response to race TTKSK and its variants will improve our understanding of the genetic value of the individual accessions. Moreover, as similar studies are completed, the genetic characterization of many different germplasm sets will provide researchers with a comprehensive global perspective of resistance to African stem rust races in the tetraploid wheat gene pool and with the possibility to transfer novel resistance genes to hexaploid wheat cultivars. The current study is amongst the first to analyse a population that represents all of the tetraploid wheat subspecies for resistance to race TTKSK at seedling stage, among which 108 durum wheat accessions are representative of the Italian durum wheat breeding programs of the last 100 years. The information that we have generated is also valuable for the choice of parental lines to use in crosses to complement genome-wide association mapping data and to analyse some of the novel associations in more detail.

## Author contributors

BS and GP carried out the phenotypic evaluations. GL and GP carried out the association mapping analysis. MR, DF, VG, and DM carried out the linkage mapping analysis. BS, GL, and AM drafted the manuscript. LC, BS, PD, and AM conceived and provided general guidance for the study. AM coordinated the study. All of the authors have critically read and approved this version of the manuscript.

## Funding

This study was supported by the Italian Ministry of Agriculture (MiPAAF), with the special grant BIOMASSVAL, ESPLORA, ISCOCEM, and CANADAIR and by the Ministry of Education, Universities and Research (MIUR), with the special grant AGROGEN. This study was supported, in part, by funds provided through a grant from the Bill and Melinda Gates Foundation and the UK Department for International Development to Cornell University for the Borlaug Global Rust Initiative Durable Rust Resistance in Wheat Project and the Lieberman-Okinow Endowment at the University of Minnesota.

### Conflict of interest statement

The authors declare that the research was conducted in the absence of any commercial or financial relationships that could be construed as a potential conflict of interest.

## Supplementary material

Figure S1 **Results of the simulation carried out in the whole collection with 10 genes whose haplotypes were designed for them to explain each around 5% variance in the MLM model (in red)**. The 10 genes were analysed in the whole collection dataset as it was characterized by an heritability very close to 0.8, and with minor allele frequency of 0.1. **(A)** Quantile-quantile plot of GLM. **(B)** Quantile-quantile plot of GLM after FDR correction. **(C)** Quantile-quantile plot of MLM.

Figure S2 **Phenotypic distribution for stem rust resistance in the whole collection, the ***durum*** sub sample and the Q2 group**.

Figure S3 **Representation of the more frequent functional classes of SNP markers mapped on the durum wheat consensus map (reported in Maccaferri et al., 2015) near to MTAs identified for stem rust race TTKSK resistance**.

Figure S4 **Allele effect plot for the marker wPt-11612 (chromosome 6A) in which the effect and the frequency are reported for the alleles “0” (absence) of Neodur and “1” (presence) of Cirillo**.

Table S1 **List of accessions of ***T. turgidum*** sub species included in the tetraploid wheat collection analyzed for seedling resistance to stem rust race TTKSK**.
