# Supplementary figures and images for: Identification of New Resistance Loci to African Stem Rust Race TTKSK in Tetraploid Wheats Based on Linkage and Genome-Wide Association Mapping
Source: Front Plant Sci. 2015 Dec 9;6:1033. doi: 10.3389/fpls.2015.01033 (PMC4673868; doi:10.3389/fpls.2015.01033)

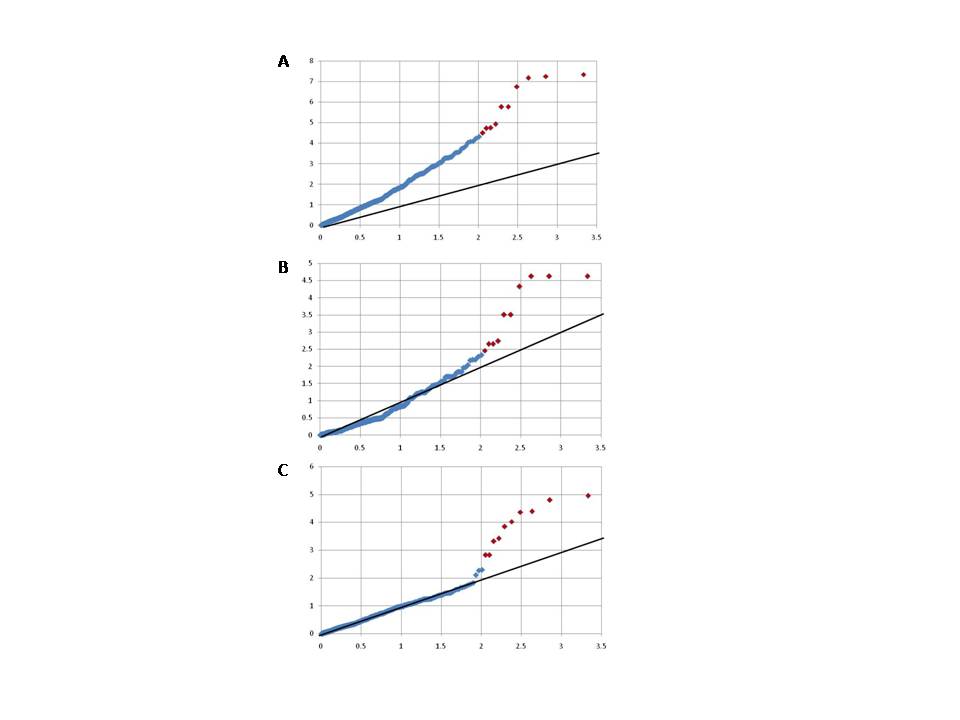

Supplement: Figure S1 — Results of the simulation carried out in the whole collection with 10 genes whose haplotypes were designed for them to explain each around 5% variance in the MLM model (in red). The 10 genes were analysed in the whole collection dataset as it was characterized by an heritability very close to 0.8, and with minor allele frequency of 0.1. (A) Quantile-quantile plot of GLM. (B) Quantile-quantile plot of GLM after FDR correction. (C) Quantile-quantile plot of MLM. [file Image1.JPEG]

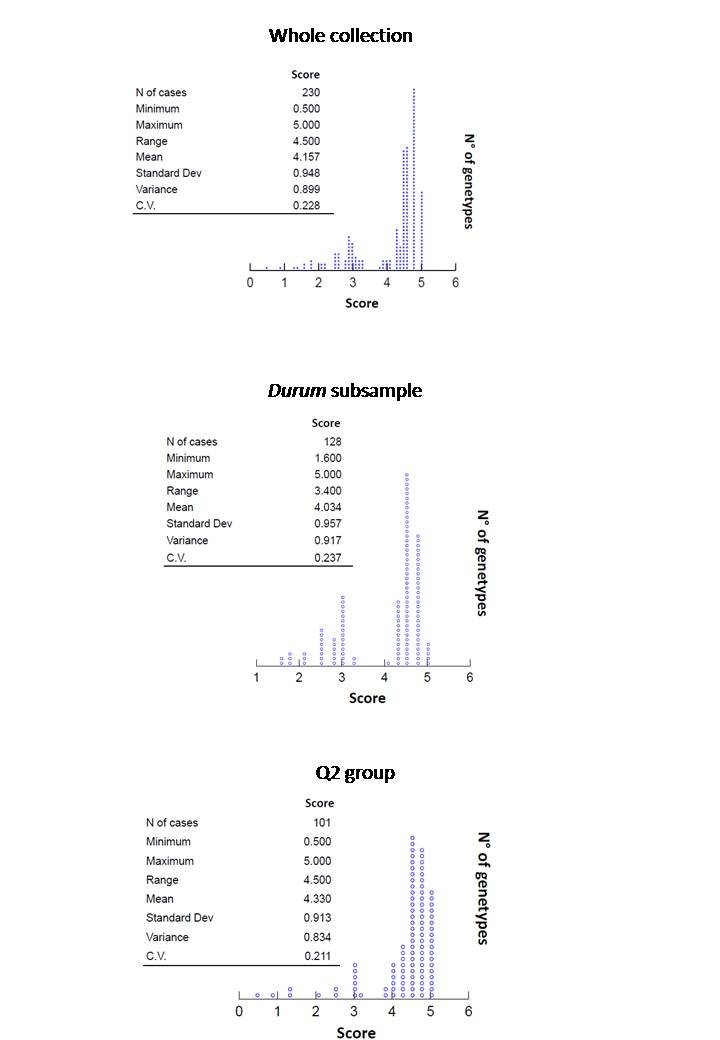

Supplement: Figure S2 — Phenotypic distribution for stem rust resistance in the whole collection, the durum sub sample and the Q2 group. [file Image2.JPEG]

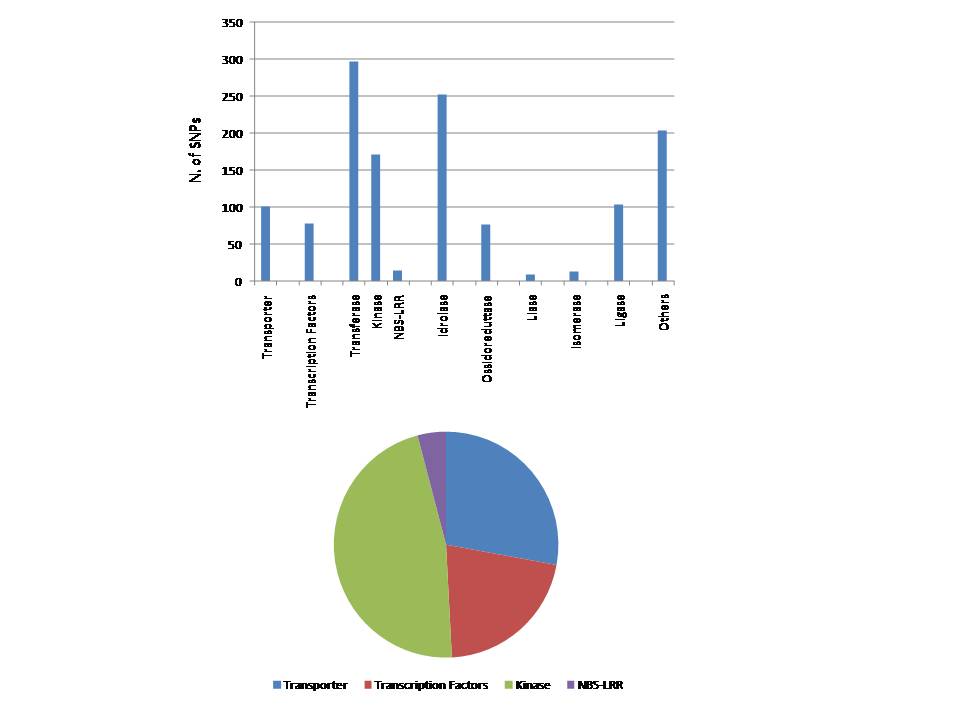

Supplement: Figure S3 — Representation of the more frequent functional classes of SNP markers mapped on the durum wheat consensus map (reported in Maccaferri et al., 2015) near to MTAs identified for stem rust race TTKSK resistance. [file Image3.JPEG]

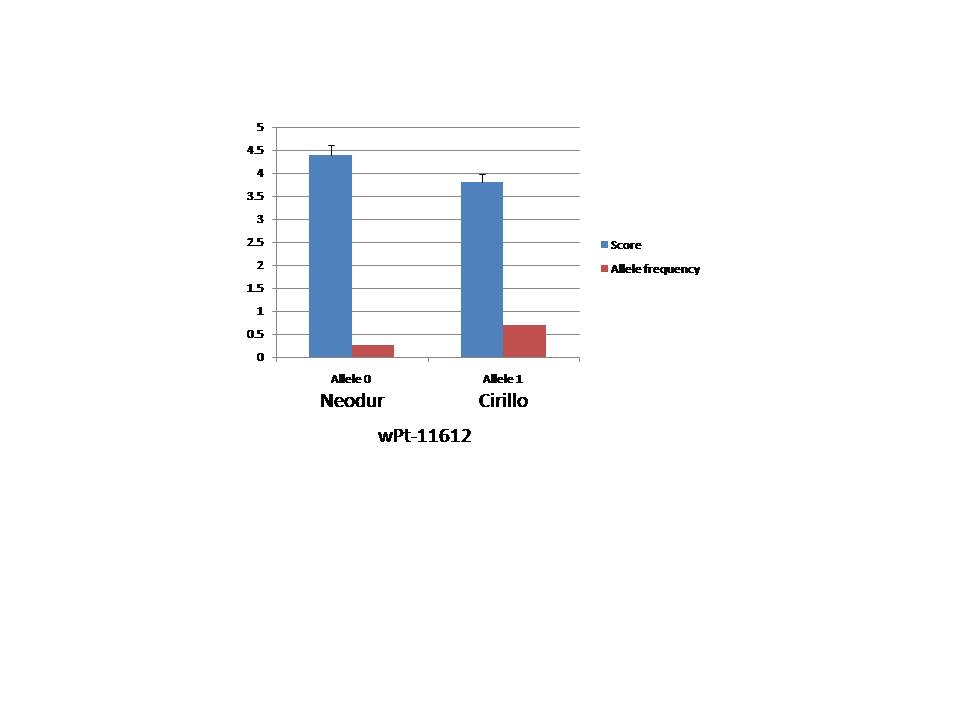

Supplement: Figure S4 — Allele effect plot for the marker wPt-11612 (chromosome 6A) in which the effect and the frequency are reported for the alleles “0” (absence) of Neodur and “1” (presence) of Cirillo. [file Image4.JPEG]
