# Supplementary material for: Identification of New Resistance Loci to African Stem Rust Race TTKSK in Tetraploid Wheats Based on Linkage and Genome-Wide Association Mapping
Source: Front Plant Sci. 2015 Dec 9;6:1033. doi: 10.3389/fpls.2015.01033 (PMC4673868; doi:10.3389/fpls.2015.01033)
Supplement: Table S1 — List of accessions of T. turgidum sub species included in the tetraploid wheat collection analyzed for seedling resistance to stem rust race TTKSK. [file Table1.DOCX]

**Table S1.** List of accessions of *T. turgidum* subspecies included in the tetraploid wheat collection analyzed for resistance at seedling stage to stem rust race TTKSK.

| **Taxonomic classification** | **Accession** | **Country** | **ITs**^a^ | **Score** |  |  | **Taxonomic classification** | **Accession** | **Country** | **ITs**^a^ | **Score** |  |
| --- | --- | --- | --- | --- | --- | --- | --- | --- | --- | --- | --- | --- |
| *T. turgidum* | 5-BIL42 | Italy | 3+ 4 | 4.8 | S |  | *T. turgidum* | PI 127106 | Afghanistan, Faryab | 4 4 | 5.0 | S |
| ssp. *durum* | Adamello | Italy | 3+ 3+ | 4.6 | S |  | ssp. *turanicum* | PI 67343 | Australia, Victoria | 4 3+ | 4.8 | S |
|  | Alemanno | Italy | 3+ 3+ | 4.6 | S |  |  | PI 68287 | Azerbaijan | 3+ 3+ | 4.6 | S |
|  | Amedeo | Italy | 3+ 3+ | 4.6 | S |  |  | PI 352514 | Azerbaijan | 4 3+ | 4.8 | S |
|  | Ancomarzio | Italy | 2 2+ | 3.0 | R |  |  | K cer | Egypt | - - | - | - |
|  | Antas | Italy | 3 3 | 4.3 | S |  |  | PI 306665 | France, Herault | 3+ 3+ | 4.6 | S |
|  | Appio | Italy | 3+ 3+ | 4.6 | S |  |  | PI 290530 | Hungary, Pest | 3+ 3+ | 4.6 | S |
|  | Appulo | Italy | 3 3 | 4.3 | S |  |  | PI 167481 | Turkey, Denizli | 3+3 | 4.5 | S |
|  | Arcangelo | Italy | 3 3 | 4.3 | S |  |  | PI 278350 | Italy | 4 3+ | 4.8 | S |
|  | Arcobaleno | Italy/Spain | 3+ 3+ | 4.6 | S |  |  | PI 623656 | Iran, West Azerbaijan | 4 3+ | 4.8 | S |
|  | Ares | Italy | 4 3+ | 4.8 | S |  |  | PI 254206 | Iran | 4 3+ | 4.8 | S |
|  | Arnacoris | Italy | 3 3 | 4.3 | S |  |  | PI 624429 | Iran, Bakhtaran | 4 3+ | 4.8 | S |
|  | Athena | Italy | 1 1- | 1.8 | R |  |  | PI 113393 | Iraq | 4 3+ | 4.8 | S |
|  | Avispa | Italy | 2 2+ | 3.0 | R |  |  | PI 191599 | Morocco, Rabat-Sale | 1 1 | 2.1 | R |
|  | Ariosto | Italy | 3 3 | 4.3 | S |  |  | PI 192641 | Morocco | 3 3- | 3.9 | S |
|  | Aziziah | Italy | 3+ 4 | 4.8 | S |  |  | PI 192658 | Morocco | 4 4 | 5.0 | S |
|  | Baio | Italy | 3+ 3+ | 4.6 | S |  |  | PI 184526 | Portugal | 2 2+ | 3.0 | R |
|  | Barcarol | Italy | 2 2+ | 3.0 | R |  |  | PI 362067 | Romania, Brasov | 3 3 | 4.3 | S |
|  | Belfuggito | Italy | 4 3+ | 4.8 | S |  |  | PI 576854 | Turkey, Diyarbakir | 3+ 4 | 4.8 | S |
|  | Berillo | Italy | 4 3+ | 4.8 | S |  |  | CItr-11390 | United States | 3- 3- | 3.3 | S |
|  | Bronte | Italy | 3+ 3+ | 4.6 | S |  |  |  |  |  |  |  |
|  | Canyon | Italy | 1 2 | 2.8 | R |  | *T. turgidum* | PI 352542 | France | 3+ 4 | 4.8 | S |
|  | Cannizzo | Italy | 4 3+ | 4.8 | S |  | ssp. *turgidum* | PI 352543 | France | 3+ 3+ | 4.6 | S |
|  | Capeiti-8 | Italy | 3+ 3+ | 4.6 | S |  |  | PI 352541 | Germany | 3 3 | 4.3 | S |
|  | Cappelli | Italy | 3+ 3+ | 4.6 | S |  |  | PI 290522 | Germany | 3+ 3+ | 4.6 | S |
|  | Casanova | Italy | 3 3 | 4.3 | S |  |  | PI 290526 | Hungary, Pest | 3+ 3+ | 4.6 | S |
|  | Chiara | Italy | 3+ 3 | 4.5 | S |  |  | PI 157983 | Italy, Sicily | 3+ 4 | 4.8 | S |
|  | Ciccio | Italy | 3+ 3+ | 4.6 | S |  |  | PI 157985 | Italy, Sicily | 3+ 4 | 4.8 | S |
|  | Ciclope | Italy | 4 4 | 5.0 | S |  |  | PI 286075 | Poland | 3 3 | 4.3 | S |
|  | Cirillo | Italy | 1-2- | 2.5 | R |  |  | PI 185723 | Portugal, Leira | 3 3 | 4.3 | S |
|  | Claudio | Italy | 3- 3- | 3.3 | S |  |  | PI 56263 | Portugal, Lisboa | 4 4 | 5.0 | S |
|  | Colosseo | Italy | 3+ 3+ | 4.6 | S |  |  | PI 134946 | Portugal, Lisboa | 4 4 | 5.0 | S |
|  | Creso | Italy | 4 3+ | 4.8 | S |  |  | PI 221423 | Portugal | 3+ 3+ | 4.6 | S |
|  | Dauno | Italy | 2 1 | 2.8 | R |  |  | PI 191104 | Spain | 2 2+ | 3.0 | R |
|  | Duilio | Italy | 4 3+ | 4.8 | S |  |  | PI 191145 | Spain, Baleares | - - | - | - |
|  | Duetto | Italy | 4 3+ | 4.8 | S |  |  | PI 191203 | Spain | 4 3+ | 4.8 | S |
|  | Dylan | Italy | 4 3+ | 4.8 | S |  |  | PI 352544 | Switzerland, Vaud | 3+ 3+ | 4.6 | S |
|  | Enduro | Italy | 2- 1 | 2.6 | R |  |  | PI 173503 | Turkey, Artvin | 4 4 | 5.0 | S |
|  | Fauno | Italy | 2- 1- | 2.5 | R |  |  | PI 341391 | Turkey, Burdur | 3+ 3+ | 4.6 | S |
|  | Fenix | Italy | 4 3+ | 4.8 | S |  |  | PI 352538 | United Kingdom, England | 3 3 | 4.3 | S |
|  | Fiore | Italy | 2 2+ | 3.0 | R |  |  |  |  |  |  |  |
|  | Fortore | Italy | 4 4 | 5.0 | S |  | *T. turgidum* | PI 352489 | Cyprus | 3+ 3+ | 4.6 | S |
|  | Gianni | Italy | 2 2+ | 3.0 | R |  | ssp. *polonicum* | PI 361757 | Denmark | 4 3+ | 4.8 | S |
|  | Giotto | Italy | 4 3+ | 4.8 | S |  |  | PI 286547 | Ecuador | 3 3 | 4.3 | S |
|  | Grazia | Italy | 1 2 | 2.8 | R |  |  | PI 366117 | Egypt, Sinai | 2 2+ | 3.0 | R |
|  | Grecale | Italy | 4 3+ | 4.8 | S |  |  | PI 387479 | Ethiopia | 2+ 2 | 3.0 | R |
|  | Grifoni | Italy | 4 3+ | 4.8 | S |  |  | PI 349051 | Georgia | 4 3+ | 4.8 | S |
|  | Hymera | Italy | 4 3+ | 4.8 | S |  |  | PI 352487 | Germany, Saxony-Anhalt | 3 3 | 4.3 | S |
|  | Imhotep | Italy | 3+ 3+ | 4.6 | S |  |  | PI 272564 | Hungary, Pest | 3- 3- | 3.3 | S |
|  | Iride | Italy | 2 2+ | 3.0 | R |  |  | PI 352488 | Italy | 3+ 3+ | 4.6 | S |
|  | Isa | Italy | 3+ 3+ | 4.6 | S |  |  | PI 210845 | Iran | 3+ 3+ | 4.6 | S |
|  | Italo | Italy | - 3- | 3.3 | S |  |  | PI 208911 | Iraq | 3 3 | 4.3 | S |
|  | K26 | Italy | 3+ 3+ | 4.6 | S |  |  | PI 223171 | Jordan | 3+ 3+ | 4.6 | S |
|  | Karel | Italy | 3+ 3+ | 4.6 | S |  |  | PI 290512 | Portugal | 3 3- | 3.9 | S |
|  | Lambro | Italy | 3+ 3+ | 4.6 | S |  |  | PI 306549 | Romania | 3 3 | 4.3 | S |
|  | Latino | Italy | 4 3+ | 4.8 | S |  |  | PI 266846 | United Kingdom, England | 4 3+ | 4.8 | S |
|  | Maestrale | Italy | 2 2+ | 3.0 | R |  |  | PI 278647 | United Kingdom, England | 3+ 3+ | 4.6 | S |
|  | Martino | Italy | 3+ 3+ | 4.6 | S |  |  | PI 289606 | United Kingdom, England | 3 3 | 4.3 | S |
|  | Meridiano | Italy | 3+ 3+ | 4.6 | S |  |  | PI 330554 | United Kingdom, England | 3+ 3+ | 4.6 | S |
|  | Messapia | Italy | 2 1 | 2.8 | R |  |  | PI 330555 | United Kingdom, England | 3+ 3+ | 4.6 | S |
|  | Mida | Italy | 3+ 3+ | 4.6 | S |  |  | PI 566593 | United States | 4 3+ | 4.8 | S |
|  | Neolatino | Italy | 3+ 3+ | 4.6 | S |  |  |  |  |  |  |  |
|  | Normanno | Italy | 2 2+ | 3.0 | R |  | *T. turgidum* | PI 94755 | Georgia | 4 3+ | 4.8 | S |
|  | Ofanto | Italy | 3+ 3+ | 4.6 | S |  | ssp. *carthlicum* | PI 115816 | Georgia | 0; 0; | 0.5 | R |
|  | Orfeo | Italy | 3 3 | 4.3 | S |  |  | PI 572849 | Georgia | 0; 1 | 1.3 | R |
|  | Orobel | Italy | 3+ 3+ | 4.6 | S |  |  | PI 499972 | Georgia | 4 3+ | 4.8 | S |
|  | PC32 | Italy | 3+ 3+ | 4.6 | S |  |  | PI 585017 | Georgia | 4 4 | 5.0 | S |
|  | Platani | Italy | 3 3 | 4.3 | S |  |  | PI 585018 | Georgia | 1-0; | 0.9 | R |
|  | Plinio | Italy | 3+ 4 | 4.8 | S |  |  | PI 283888 | Iran | 3+ 3+ | 4.6 | S |
|  | Polesine | Italy | 3+ 3+ | 4.6 | S |  |  | PI 70738 | Iraq | 4 4 | 5.0 | S |
|  | PR22D89 | Italy | 3+ 3+ | 4.6 | S |  |  | PI 341800 | Russian Federation, Dagestan | 4 4 | 5.0 | S |
|  | Preco | Italy | 3+ 3+ | 4.6 | S |  |  | CItr 7665 | Russian Federation | 3+ 3+ | 4.6 | S |
|  | Provenzal | Italy | 1 1 | 2.1 | R |  |  | PI 532501 | Former Soviet Union | 4 4 | 5.0 | S |
|  | Quadrato | Italy | 4 4 | 5.0 | S |  |  | PI 573182 | Turkey, Kars | 3+ 3 | 4.5 | S |
|  | Quadruro | Italy | 3+ 4 | 4.8 | S |  |  |  |  |  |  |  |
|  | Russello | Italy | 3 3 | 4.3 | S |  | *T. turgidum* | ISC Foggia 171 | Ethiopia | 2 2+ | 3.0 | R |
|  | Rusticano | Italy | 1 2 | 2.8 | R |  | ssp. *dicoccum* | MG 5350 | Ethiopia | 3+ - | 4.6 | S |
|  | Taganrog | Italy | 4 3+ | 4.8 | S |  |  | MG 5344/1 | Ethiopia | 3+ 3+ | 4.6 | S |
|  | Timilia | Italy, Sicily | 3+ 3+ | 4.6 | S |  |  | ISC Foggia 175 | Hungary | 4 4 | 5.0 | S |
|  | Tito | Italy | 4 3+ | 4.8 | S |  |  | MG 5293/1 | Italy | 4 4 | 5.0 | S |
|  | Tiziana | Italy | 1- 1 | 1.8 | R |  |  | Farvento | Italy | 4 3+ | 4.8 | S |
|  | Tresor | Italy | 2 2+ | 3.0 | R |  |  | Lucanica | Italy | 4 3+ | 4.8 | S |
|  | Trinakria | Italy | 3+ 3+ | 4.6 | S |  |  | Molise sel. Colli | Italy | 3+ 3+ | 4.6 | S |
|  | San Carlo | Italy | 4 4 | 5.0 | S |  |  | ISC Foggia 152 | Iran | 3+ 3+ | 4.6 | S |
|  | Sansone | Italy | 4 3+ | 4.8 | S |  |  | MG 5416/1 | Iran | 4 4 | 5.0 | S |
|  | Saragolla | Italy | 1- 2- | 2.5 | R |  |  | ISC Foggia 159 | Morocco | 3+ 3+ | 4.6 | S |
|  | Simeto | Italy | 1 1+ | 2.2 | R |  |  | MG 5471/1 | Spain | 4 4 | 5.0 | S |
|  | Svevo | Italy | 2 2+ | 3.0 | R |  |  | MG 5473 | Spain | 4 3+ | 4.8 | S |
|  | Valforte | Italy | 3+ 3+ | 4.6 | S |  |  | MG 15516/1 | Syria | 3 3 | 4.3 | S |
|  | Valgerardo | Italy | 3+ 3+ | 4.6 | S |  |  | ISC Foggia 161 | United Kingdom | 4 4 | 5.0 | S |
|  | Valnova | Italy | 4 3+ | 4.8 | S |  |  | MG 4387 | United Kingdom | 4 3+ | 4.8 | S |
|  | Varano | Italy | 3+ 3+ | 4.6 | S |  |  | MG 5323 | n.a. | 2 2+ | 3.0 | R |
|  | Vendetta | Italy | 4 3+ | 4.8 | S |  |  | MG 3521 | n.a. | 4 3+ | 4.8 | S |
|  | Vesuvio | Italy | 3 3 | 4.3 | S |  |  | MG 5300/1 | n.a. | - - | - | - |
|  | Vitromax | Italy/Spain | 3 3 | 4.3 | S |  |  |  |  |  |  |  |
|  | Zenit | Italy | 1 1 | 2.1 | R |  | *T. turgidum* | PI 355459 | Armenia | 3 3 | 4.3 | S |
|  | Agridur | France | 3 3 | 4.3 | S |  | ssp. *dicoccoides* | PI 352323 | Asia Minor | 3 3 | 4.3 | S |
|  | Ambral | France | 2 2+ | 3.0 | R |  |  | PI 346783 | Hungary, Pest | 4 4 | 5.0 | S |
|  | Brindur | France | 4 3+ | 4.8 | S |  |  | PI 343446 | Israel | 3 3- | 3.9 | S |
|  | Ceedur | France | 3+ 3+ | 4.6 | S |  |  | PI 481539 | Israel | 3+ - | 4.6 | S |
|  | Cosmodur | France | 2 2+ | 3.0 | R |  |  | PI 352324 | Lebanon | 3 3 | 4.3 | S |
|  | Doral | France | 4 3+ | 4.8 | S |  |  | PI 470944 | Syria, Al Qunaytirah | 3 3 | 4.3 | S |
|  | Durfort | France | 1 2- | 2.6 | R |  |  | PI 470945 | Syria, Al Qunaytirah | 4 3+ | 4.8 | S |
|  | Exeldur | France | 3 3 | 4.3 | S |  |  | MG 4343 | n.a. | 4 4 | 5.0 | S |
|  | Latinur | France | 4 4 | 5.0 | S |  |  | MG 4328/61 | n.a. | 4 4 | 5.0 | S |
|  | Nefer | France | 3+ 3+ | 4.6 | S |  |  | MG 5444/235 | n.a. | 4 3+ | 4.8 | S |
|  | Neodur | France | 3+ 3+ | 4.6 | S |  |  | MG 4330/66 | n.a. | 4 4 | 5.0 | S |
|  | Parsifal | France | 3+ 3+ | 4.6 | S |  |  |  |  |  |  |  |
|  | Primadur | France | 1 0; | 1.3 | R |  |  |  |  |  |  |  |
|  | Saadi | France | 4 3+ | 4.8 | S |  |  |  |  |  |  |  |
|  | Virgilio | France | 2- 1- | 2.5 | R |  |  |  |  |  |  |  |
|  | Kronos | United States | 4 3+ | 4.8 | S |  |  |  |  |  |  |  |
|  | Langdon | United States | - - | - | - |  |  |  |  |  |  |  |
|  | Lloyd | United States | 3+ 3+ | 4.6 | S |  |  |  |  |  |  |  |
|  | L092 | United States | 2 2+ | 3.0 | R |  |  |  |  |  |  |  |
|  | L252 | United States | 3+ 3+ | 4.6 | S |  |  |  |  |  |  |  |
|  | Produra | United States | 3 3 | 4.3 | S |  |  |  |  |  |  |  |
|  | S99B34 | United States | 3+ 3+ | 4.6 | S |  |  |  |  |  |  |  |
|  | West Bread 881 | United States | 0; 1 | 1.3 | R |  |  |  |  |  |  |  |
|  | AC-Navigator | Canada | 2 1 | 2.8 | R |  |  |  |  |  |  |  |
|  | Strongfield | Canada | 2 2+ | 3.0 | R |  |  |  |  |  |  |  |
|  | UC1113 | Canada | 3 3+ | 4.5 | S |  |  |  |  |  |  |  |
|  | Altar84 | Mexico | 2- 1- | 2.5 | R |  |  |  |  |  |  |  |
|  | Mexicali 75 | Mexico | 3+ 4 | 4.8 | S |  |  |  |  |  |  |  |
|  | Granizo | Spain | 2- 1- | 2.5 | R |  |  |  |  |  |  |  |
|  | Pedroso | Spain | 1- 1 | 1.8 | R |  |  |  |  |  |  |  |
|  | Sharm 5 | Syria | 3+ 3+ | 4.6 | S |  |  |  |  |  |  |  |
|  | Kyperounda | Morocco | 3+ 3+ | 4.6 | S |  |  |  |  |  |  |  |

^a^ Infection types (ITs) observed on seedlings at 14 days post inoculation using a 0-to-4 scale according to Stakman et al. (1962), where ITs of 0, ;, 1, 2, or combinations are considered

to be low ITs and ITs of 3 or higher are considered to be high. "n.a."denotes that the information is not available; and - denotes missing data.
